# K_Ca_2 and K_Ca_3.1 Channels in the Airways: A New Therapeutic Target

**DOI:** 10.3390/biomedicines11071780

**Published:** 2023-06-21

**Authors:** Razan Orfali, Ali AlFaiz, Mohammad Asikur Rahman, Liz Lau, Young-Woo Nam, Miao Zhang

**Affiliations:** 1 Department of Biomedical and Pharmaceutical Sciences, Chapman University School of Pharmacy, Irvine, CA 92618, USA; rorfali@kfmc.med.sa (R.O.);; 2 Biomedical Research Administration, Research Centre, King Fahad Medical City, Riyadh Second Health Cluster, Riyadh 12231, Saudi Arabia

**Keywords:** K_Ca_2 channels, lungs, motile cilia, cystic fibrosis, anosmia, chronic obstructive pulmonary diseases

## Abstract

K^+^ channels are involved in many critical functions in lung physiology. Recently, the family of Ca^2+^-activated K^+^ channels (K_Ca_) has received more attention, and a massive amount of effort has been devoted to developing selective medications targeting these channels. Within the family of K_Ca_ channels, three small-conductance Ca^2+^-activated K^+^ (K_Ca_2) channel subtypes, together with the intermediate-conductance K_Ca_3.1 channel, are voltage-independent K^+^ channels, and they mediate Ca^2+^-induced membrane hyperpolarization. Many K_Ca_2 channel members are involved in crucial roles in physiological and pathological systems throughout the body. In this article, different subtypes of K_Ca_2 and K_Ca_3.1 channels and their functions in respiratory diseases are discussed. Additionally, the pharmacology of the K_Ca_2 and K_Ca_3.1 channels and the link between these channels and respiratory ciliary regulations will be explained in more detail. In the future, specific modulators for small or intermediate Ca^2+^-activated K^+^ channels may offer a unique therapeutic opportunity to treat muco-obstructive lung diseases.

## 1. Introduction

The epithelial surface of the respiratory tract between the nose and the alveoli is constantly exposed to potentially harmful pathogens, particulates, and gaseous materials [1,2,3]. In response to these challenges, the human body utilizes a series of defense mechanisms to protect the airways, and the primary defense mechanism in the lung is mucociliary clearance (MCC) [1,4]. MCC is a process of specialized organelles called cilia that beat in metachronal waves to impel pathogens and particles trapped by the mucous layer out of the airways. Cilia within the mucociliary system present critical functions in human health; abnormalities in each compartment of the mucociliary system could compromise the mucus clearance process and lead to chronic lung disease [2,3]. Mucociliary dysfunction is commonly associated with chronic airway diseases, and it is one of the pathological observations in patients with cystic fibrosis, primary ciliary dyskinesia, chronic bronchitis, and asthma [5,6]. Airway diseases with associated mucociliary dysfunction remain largely unaddressed, despite the therapeutic progress in treating inflammatory lung diseases [5].

The lung’s lining is covered by a thin layer of fluid called airway surface liquid (ASL); it separates the airway epithelium’s luminal surface from the external environment. ASL is mainly composed of water, electrolytes, and mucins; it is essential for normal airway function, particularly for proper MCC [3,7,8]. ASL epithelia contain various cell types with distinct morphologies and functions. Of the cell population in the trachea, approximately 60% are ciliated cells; these cells also retain other important roles other than coordinating ciliated movements, such as regulating ion transfer [1,9].

There are detections of over 30 diverse K^+^ channels in the airway epithelia, and these K^+^ channels maintain the electrochemical gradient and support lung ion and fluid homeostases [1,10,11,12]. A large portion of airway chloride secretion occurs through the apically located bicarbonate and chloride channels [10]. K^+^ channels are involved in many vital functions in lung physiology, such as oxygen sensing, inflammatory responses, enhancing Cl^−^ transport, and respiratory epithelia repair [9,13]. The basolateral K^+^ channel has known regulation effects on Na^+^ absorption; reduced Na^+^ absorption in the lung shows improvement in muco-obstructive disease. A large portion of airway chloride secretion occurs through the apically located bicarbonate and chloride channels, significantly influenced by some Ca^2+^-activated K^+^ channels (K_Ca_) that are located apically in the lung [10]. Hence, the specific K^+^ group K_Ca_ also regulates MCC and ASL volumes [1]. The small-conductance K_Ca_2 channels and intermediate-conductance K_Ca_3.1 channels are voltage-independent and activated solely by the elevation of the intracellular Ca^2+^ concentration. In this context, we will discuss current knowledge of the functional roles of K_Ca_2 and K_Ca_3.1 channels in the respiratory tract, focusing on their physiological roles in respiratory diseases.

## 2. Introduction to K_Ca_ Channels

There are several kinds of K^+^ channels present in the respiratory epithelium lining airways, and the most indispensable K^+^ channels in airway epithelial cells are the Ca^2+^-activated K^+^ channels. They serve as the cell crossroad where Ca^2+^ influx, other ion outfluxes, and membrane potential, all processes governed by K_Ca_ channels, integrate to modulate an extensive array of cellular processes [14]. K_Ca_ channels are subdivided into three major groups, according to their single-channel conductance: large conductance (150–300 pS) K^+^ channels (BK or K_Ca_1.1), small conductance (2–20 pS) K^+^ channels (SK or K_Ca_2), and intermediate conductance (20–60 pS) K^+^ channels (IK or K_Ca_3.1) [15,16,17]. Each group has specific distinct biophysical and pharmacological properties [18]. K_Ca_2.x and K_Ca_3.1 channels are voltage-independent and activated exclusively by intracellular Ca^2+^ via the calmodulin (CaM) that is typically bound to these channels and serves as their Ca^2+^ sensor [19]. K_Ca_2x and K_Ca_3.1 channels, before their cloning, were referred to as small-conductance (SK) or intermediate-conductance (IK) Ca^2+^-activated K^+^ channels, based on their singular conductance of ~10 pS or ∼40 pS in symmetrical solutions to differentiate them from the large-conductance potassium (BK) channel [19,20].

Four *mammalian KCNN* channel subtypes are encoded by the *KCNN* genes, including *KCNN1* for K_Ca_2.1, *KCNN2* for K_Ca_2.2, *KCNN3* for K_Ca_2.3 [21], and *KCNN4* for K_Ca_3.1 [22], respectively [23] (Table 1).

### K_Ca_2 and K_Ca_3.1 Channel Structures

**Figure 1:**
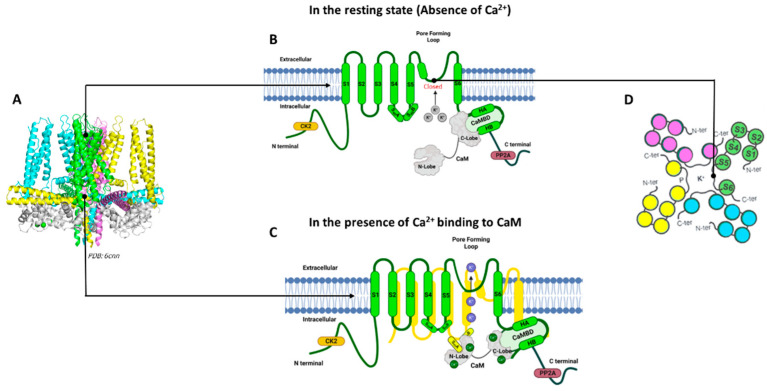
**K_Ca_3.1 and K_Ca_2 Channel Structures in the presence and absence of Ca^2+^.** K_Ca_2 and K_Ca_ 3.1 channels are assembled as homotetramers of four α-subunits. (**A**) Human K_Ca_3.1 channel cryo-EM structure (PDB: 6cnn). For clarity, four-channel subunits are shown in different colors: green, blue, yellow, and purple, along with calmodulin (CaM) (gray). (**B**) Schematic representation of one channel subunit in the absence of Ca^2+^. (**C**) Schematic representation of one channel subunit in the presence of Ca^2+^. (**D**) Extracellular top view of the K_Ca_3.1 and K_Ca_2 channels. (**A**) was generated using Biorender.com. (**B**,**C**) were generated using Pymol (Schrödinger, LLC, New York, NY, USA).

K_Ca_2 and K_Ca_3.1 channels are assembled as homotetramers of four α-subunits; each subunit is composed of six transmembrane α-helical domains denoted as S1–S6 (Figure 1). The selectivity filter within the channel pore between the S5 and S6 transmembrane domains is responsible for the selective permeability of the K^+^ ions [30,34]. The K_Ca_2/K_Ca_3.1 channel subtypes are highly homologous in their six transmembrane domains, but the amino acid sequences and lengths at their cytoplasmic N- and C-termini differ among the subtypes (Table 1) [37].

Among the four K_Ca_2/K_Ca_3.1 channel subtypes, the full-length cryogenic electron microscopy (cryo-EM) structure is only available for the K_Ca_3.1 channel determined in the absence and presence of Ca^2+^, providing insight into the Ca^2+^/CaM gating mechanism for these channels [36]. The calmodulin-binding domain consists of two α-helices, HA and HB, whereas the S4–S5 linker includes two α-helices, S_45_A and S_45_B. The HA and HB helices from one channel subunit, the S4–S5 linker from a neighboring channel subunit, and calmodulin closely interact with each other (Figure 1). When Ca^2+^ is absent, the C-lobe of CaM binds to the HA/HB helices in the proximal channel C-terminus, the N-lobe of CaM is highly flexible, and the channel pore is closed (Figure 1B). In the presence of Ca^2+^, the N-lobe of CaM becomes well-structured and interacts with the linker between the S4 and S5 transmembrane domains (S4–S5 linker) of a neighboring α-subunit. The interaction between the Ca^2+^-bound CaM N-lobe and the S4–S5 linker causes the movement of the S6 transmembrane domain and the opening of the channel pore (Figure 1C) [33,38].

K_Ca_2 channels are activated by Ca^2+^, with EC_50_ values ranging from 300 to 750 nM, whereas K_Ca_3.1 channels exhibit apparent Ca^2+^ sensitivities of 100–400 nM [29,34,39]. K_Ca_2 and K_Ca_3.1 channels, therefore, play a critical role in the physiologies of various tissues and disease states [22,40]. The advances in understanding the K_Ca_3.1 structure [36] (the cryo-electron microscopy of the human homotetrameric *KCNN4* channel) and the resulting improvements in other K_Ca_2 subtypes modeling [29,30,34] have yet to be used, not only for drug discovery but also for understanding the pathophysiological diseases.

## 3. K_Ca_ Channels in the Respiratory System

The involvement of K^+^ channels has been proposed in respiratory conditions such as asthma, chronic obstructive pulmonary diseases (COPD), and cystic fibrosis (CF) [1,12]. In airway epithelial cells, both Cl^−^ and K^+^ transports rely, to some extent, on Ca^2+^-dependent channel activity (e.g., K_Ca_ channels) [1]. K_Ca_ channels are important in regulating Cl^−^ secretion, MCC, and ASL volumes. K_Ca_3.1 and K_Ca_2 channel subtypes located in the airway epithelia, such as K_Ca_2.1 [41] and K_Ca_2.3 [42], maintain the electrochemical gradient and thus support lung ion and fluid homeostasis [1]. Table 2 summarizes the *KCNN* gene family, tissue distribution, physiological roles, and their roles in the lungs.

### 3.1. K_Ca_ Channels and the Respiratory Cilia

The K_Ca_2 and K_Ca_3.1 channels are tetramers, and each subunit comprises six transmembrane alpha-helical domains (six TMD), indicated as S1–S6 in each channel subunit. The selectivity of potassium ions across these channels is based on the pore-forming P-loop between the transmembrane S5 and S6 domains. K_Ca_2/K_Ca_3.1 are more sensitive to Ca^2+^ due to calmodulin CaM acting as a Ca^2+^ sensor (Figure 1) [26,47]. CaM is present in all eukaryotic cells, facilitating various cellular signaling processes, such as the modulation of ion channel actions, regulation of enzymatic activities, and gene expression [14,48]. The ciliary beat of the airway epithelium is believed to be regulated by the level of intracellular Ca^2+^ [49]. The association with calmodulin in the regulation of ciliary beats has been reported as the most important intraciliary Ca^2+^ binding protein [49,50]. Moreover, the activation of K_Ca_2 channels in non-excitable cells, such as epithelial cells, increases Ca^2+^ entry through non-voltage-gated Ca^2+^ channels, thereby increasing intracellular Ca^2+^ concentration [51]. This elevation of intracellular Ca^2+^ is one of the primary regulators of ciliary movement [52]. Thus, K_Ca_2 and K_Ca_3.1 channels will regulate respiratory ciliary activities as part of a complex signaling network.

#### 3.1.1. K_Ca_ Channels and Ciliary Beat Frequency

In vitro measurements of the changes in the CBF of human respiratory cells indicate that Ca^2+^ ionophore speeds the CBF of human respiratory cells mediated through a calmodulin-sensitive system [53]. Airway epithelial cells contain 100 nM of free Ca^2+^ in their cytoplasm, but ciliated cells bear a higher concentration at baseline than club cells [54]. This supports the idea that K_Ca_2 channels may be active during normal conditions in specific airway cells, as these channels show a high sensitivity to Ca^2+^ (Table 2). Significantly, in CF mouse airways, a previous study by Vega et al. [5] determined that *KCNN4*-silencing enhanced MCC when Na^+^ absorption was decreased. Additionally, CBF was also increased by K_Ca_3.1 inhibition. An explanation is that K_Ca_3.1 inhibition reduces Na^+^ absorption in CF, thereby increasing CBF speeds by hyperpolarizing the apical membrane [5,55].

#### 3.1.2. K_Ca_2 Channels and Cilium Length

Muco-obstructive lung disease is considered the primary cause of morbidity and is responsible for 80% of mortality [55]. The presence of K_Ca_2 channels in a human bron-chial epithelial cell, and structural similarities in the groups of K_Ca_2 and K_Ca_3.1, pro-vides a new direction in the investigating the expression and function of K_Ca_2 channel subtypes in the ciliated human lung epithelial cells. Optimal MCC requires mucus, cilia, and a thin layer of ASL to facilitate ciliary beating [2]. Maintaining a normal range of respiratory cilia length (4 to 7 μm, depending on the airway region) is critical for adequate mucociliary clearance [56]. A qualitative difference exists between short and longer cilia waveform shapes [57], and various acquired lung disorders are marked by abnormalities in both cilia structure and function [56]. Our previous work determined the critical role of K_Ca_2.3 channels in regulating the primary cilia in endothelial cells [58]. Taking advantage of the previous results could help to connect K_Ca_2 channels and respiratory cilia, two crucial components in the Ca^2+^ signaling network of airway epithelial and smooth muscle cells, with potential implications in the pathogenesis of airway diseases.

## 4. Expression and Physiological Functions of K_Ca_2 and K_Ca_3.1 Channels in the Airways

Many human cells express K_Ca_ channels that have the exceptional ability to trans-late changes in the level of the intracellular second messenger, Ca^2+^, to changes in membrane K^+^ conductance and, thus, resting potential membrane. While K_Ca_ channel subtypes are all regulated by intracellular Ca^2+^, they are otherwise quite distinct entities, differing in tissue distribution and functions [59]. K_Ca_2 channel subtypes, for example, are widely expressed in the nervous system, where they are involved in regulating the firing frequency of various neurons. On the other hand, the K_Ca_3.1 channel subtype is expressed in peripheral cells, including the erythrocytes and lymphocytes, and has been determined in numerous cancer cells where they have been implicated in growth control [60,61]. Here we demonstrate the expressions and physiological roles of K_Ca_2 and K_Ca_3.1 channels in the airways.

### 4.1. Expression and Functions of K_Ca_2 in the Respiratory Epithelia

**Figure 2:**
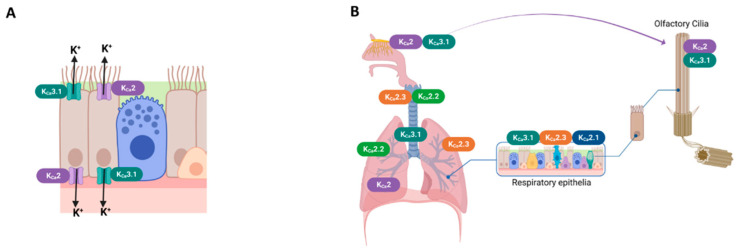
**Expression sites of K_Ca_2 and K_Ca_3.1 channels in the respiratory system**. (**A**) Schematic drawing of ciliated airway epithelial cells of K_Ca_2 and K_Ca_3.1 channels. (**B**) K_Ca_2 and K_Ca_3.1 channels were expressed in airway smooth muscle [65], airway olfactory nerves [66] and olfactory cilia [64]; K_Ca_2.2 and K_Ca_2.3 subtypes presented in the lungs and the trachea [45]; and K_Ca_3.1, K_Ca_2.1, and K_Ca_2.3 subtypes presented in the respiratory epithelia [41,42]. The pulmonary artery expressed the K_Ca_2.3 subtype [42].

K_Ca_2 channels are widely expressed in various tissues and play an important role in modulating excitable and non-excitable cells. The presence of K_Ca_ channel groups was confirmed at the apical and basolateral membranes of airway epithelial cells [1,62] (Figure 2A). The bronchial epithelium expresses K_Ca_2.1 and K_Ca_2.3 channel subtypes[35,41]. K_Ca_2.2 and K_Ca_2.3 mRNA were detected in the lungs and trachea [3,4]. K_Ca_2.2 and K_Ca_2.3 mRNA were detected in lungs and trachea [6]. K_Ca_2.3 is the only subtype expressed in the pulmonary artery [5]. Figure 2-B shows the major expression sites of K_Ca_2 and K_Ca_3.1 channel subtypes in the airway.

Different ion channels seem to be present in motile cilia [63]. In the nasal cavity, olfactory receptor neurons (ORNs) are adapted to grow various long cilia; they are not motile but can move with the liquid stream of the nasal mucosa to sample odorants entering the nose. The presence of K_Ca_ channel groups in the cilia of ORNs was reported [64].

The involvement of K^+^ channels has been proposed in respiratory conditions such as asthma, chronic obstructive pulmonary diseases (COPD), and cystic fibrosis (CF) [1,12]. In airway epithelial cells, both Cl^−^ and K^+^ transports rely, to some extent, on Ca^2+^-dependent channel activity (e.g., K_Ca_ channels) [1]. K_Ca_ channels are important in regulating Cl^−^ secretion, MCC, and ASL volumes. K_Ca_2 channel subtypes located in the airway epithelia, such as K_Ca_2.1 [41] and K_Ca_2.3 [42], maintain the electrochemical gradient and thus support lung ion and fluid homeostases [1].

In CF, the equilibrium between Na^+^ absorption and Cl^−^ secretion throughout the airway epithelia is necessary to maintain adequate ASL volume and MCC. The Cl^−^ secretion in the lungs involves several steps, starting from Cl^−^ entry through a basolateral channel cotransporter, followed by its exit via apical Cl^−^ channels, such as the cystic fibrosis transmembrane conductance regulator (CFTR) [3]. The dysfunction of CFTR channels in CF results in decreased Cl^−^ and fluid secretions and increased Na^+^ absorption, leading to inefficient mucociliary clearance and mucus accumulation [67].

In COPD, K_Ca_ channel groups can also act as oxygen sensors for lung diseases, such as COPD associated with pulmonary hypertension [12]. In COPD, pulmonary hypertension is generally believed to be due to hypoxic pulmonary vasoconstriction [68]. K_Ca_ channels potentiated by the low partial pressure of oxygen (PO_2_) have been investigated in cerebral resistance myocytes [69]. When hypoxia occurs, K_Ca_ channels activate (preventing repolarization) and relax the pulmonary arteries [70,71].

K_Ca_ channels were proposed as new targets for bronchodilator therapy for chronic diseases such as asthma and COPD [72]. The mentioned COPD-related studies [69,70,71,72] examined K_Ca_ channel groups in general. Though one study suggested that human pulmonary artery and bronchial relaxations might be mediated by pharmacological activation of the K_Ca_2.3 channel subtypes [42] (Table 2).

In anosmia, Odorant-induced K^+^ conductance is activated by Ca^2+^ [73], and the elevation of intracellular Ca^2+^ is often associated with odorant stimulation in some vertebrates and human olfactory neurons [51,74]. Olfactory Receptor Neurons (ORNs) are located in the nasal epithelia and exhibit spontaneous action potential firing. All K_Ca_ channel groups have been detected in olfactory cilia [52], and the electrophysiological of the whole-cell results confirmed that K_Ca_ channels participate in inhibitory chemo-transduction in the cilia [75]. According to these findings, an apical Ca^2+^ influx opens the K_Ca_ channels, causing membrane hyperpolarization in response to Ca^2+^ influx and thus triggering the inhibition [74].

Moreover, a Ca^2+^ channel blocker, nifedipine, was tested on odorants that induce an inhibitory current in olfactory neurons [74]. This drug effectively abolished the outward current and stimulated the cells with an odorant solution free of nifedipine, and the response was restored [74]. K_Ca_2 channel subtypes which are completely Ca^2+^-dependent and voltage-independent may play a critical role in treating certain diseases, given the drugs that could target specific ion channels. For example, anosmia could be treated by targeting the K_Ca_2 channel subtypes in olfactory cilia and testing their allosteric modulators [52]. However, the pharmacology of these channels in olfactory neurons has not been fully characterized.

### 4.2. Expression and Functions of K_Ca_3.1 in the Respiratory Epithelia

The expression of Ca^2+^-activated potassium (K_Ca_) channels often correlate positively with cell proliferation. As an example, the expression of K_Ca_3.1 increases 4-fold upon T-lymphocyte activation, and this channel is inhibited with the specific inhibitor that inhibits T-lymphocyte proliferation [76]. This is because the K_Ca_3.1 channel contributes to electrochemical gradients for Ca^2+^ influx, which is critical for the proliferation of the T cells [77]. K_Ca_3.1 is also broadly expressed in other cells of the immune system, such as B cells, macrophages, microglia, and mast cells [78]. The major function of K_Ca_3.1 in immune cells is to hyperpolarize the cell membrane and create the driving force for calcium entry, which is necessary for proliferation, activation, and cytokine production [79]. Previous findings [80] suggest that antigen sensitization up-regulates K_Ca_3.1 expression, which may contribute to enhancing cell migration in response to lymphatic chemokines, particularly in the immunogenic lung dendritic cells subset. Therefore, targeting K_Ca_3.1 crucial for controlling allergic airway inflammation [81] (Table 2 and Figure 2).

K_Ca_ channels have been found to be involved in regulating smooth muscle responses to both contractile and relaxant agonists that elevate intracellular Ca^2+^ [82]. Phenotypic modulation of smooth muscle cells is accompanied by changes in K_Ca_3.1 channel expression characterizing “proliferative” cells [83]. K_Ca_3.1 channels regulate the proliferative responses of vascular smooth muscle cells, fibroblasts, endothelial cells, and T lymphocytes, as well as a some transformed cell types [61,84]. K_Ca_3.1 function is increased by protein kinase A (PKA) [85] and nucleoside diphoshate kinase B (NDPK-B) and inhibited by the histidine phosphatase PHPT1 [86,87]. Since NDPK-B and PHPT1 directly phosphorylate or dephosphorylate K_Ca_3.1 on histidine in the C-terminus, K_Ca_3.1 modulation in mammals is one of the rare examples of histidine kinase/phosphatase regulating a biological process [86].

In allergic lung diseases, K_Ca_3.1 channels regulate Ca^2+^ entry into cells and thereby modulate Ca^2+^-signaling processes. The entry of positively charged Ca^2+^ into the cells depolarizes the membrane, which limits its own ability to enter the cell through some types of Ca^2+^ channels that are closed at more positive membrane potentials. K_Ca_3.1 activation by elevated intracellular Ca^2+^ maintains a negative membrane potential, which helps to sustain Ca^2+^ entry into the cell. K_Ca_3.1-mediated elevation of intracellular Ca^2+^ is necessary for the production of inflammatory chemokines and cytokines by T cells, mast cells, and macrophages [79,88]. Indeed, proliferation is accompanied by the transcriptional up-regulation of functional K_Ca_3.1 expression and can be inhibited by K_Ca_3.1 inhibitors [86]. It has been reported that the use of K_Ca_3.1 blockers can provide a potential therapeutic target for mast cell-mediated diseases such as asthma [88]. Moreover, blocking K_Ca_3.1 may offer a novel approach to treating idiopathic pulmonary fibrosis [89].

In muco-obstructive hyper tension, the inhibition of the K_Ca_3.1 channel [5] and *Kcnn4* silencing in ion transport and MCC in an animal model of CF/COPD-like muco-obstructive lung disease determined that *Kcnn4* silencing enhances airway disease [5]. The effectiveness of the mucociliary clearance depends mainly on hydration. Water availability in the airways is controlled by transepithelial ion transport. Apical Cl^−^ secretion and Na^+^ absorption play major roles in ASL volume homeostasis [90]. The decline in Na^+^ absorption is of potential benefit in muco-obstructive disorders, such as cystic fibroses. It was described earlier in the case of the kidney and intestine, where the inhibition of basolateral K^+^ channels decreased Na^+^ absorption [5,57], thus supporting the role of K^+^ channels on epithelial Na^+^ homeostasis. 

In pulmonary artery hypertension, elevated pulmonary artery pressure occurs in several diseases, such as asthma, end-stage chronic obstructive pulmonary disease (COPD), and lung fibrosis [66,91,92]. In order to diagnose pulmonary artery hypertension, hemodynamic measurements are taken via right heart catheterization or echocardiography; the condition is defined as a mean pulmonary artery pressure above 25 mmHg at rest or greater than 30 mmHg during normal physical activity [92]. Studies suggest that pharmacologically activating K_Ca_3.1 channels mediates human pulmonary artery and bronchial relaxations [42]

## 5. Pharmacological K_Ca_2 and K_Ca_3.1 Channel Modulators in Respiratory Diseases

The K_Ca_2.3 and K_Ca_3.1 potassium channels are characterized by their voltage independence, and thus, they are activated by intracellular Ca^2+^. Due to the distinct distribution of the channel subtypes in the mammalian cells and their involvement in the generation of afterhyperpolarization currents, there has been considerable interest in developing subtype-selective pharmacological tools to study these channels [93,94]. Additionally, K_Ca_2.3 and K_Ca_3.1 channels comprise attractive new targets for several diseases that currently have no effective therapies. The pharmacology of K_Ca_ channels developed relatively rapidly after the cloning of the K_Ca_2 and K_Ca_3.1 channels, as the field now has a wide range of peptides, small-molecule inhibitors, and positive- and negative-gating modulators with differential subtype selectivity available [44].

The K_Ca_3.1 and K_Ca_2 channels have relatively well-developed pharmacological tools. The field now has a wide range of peptides, small-molecule inhibitors, and positive- and negative-gating modulators with differential subtype selectivity available [93]. Table 3 shows the small molecule positive and negative modulators with differential K_Ca_2 subtype selectivity [44]. For treating CF and other mucociliary diseases, K_Ca_3.1 inhibitors are needed [5]. Senicapoc [95] and TRAM-34 [96] inhibit K_Ca_3.1 channels with IC_50_ values of ~11 nM, and ~20 nM, respectively, and they are highly selective for K_Ca_3.1 channels over K_Ca_2 channel subtypes [33]. The selective negative modulator for the K_Ca_2 channel AP14145 is equipotent in inhibiting K_Ca_2.2 and K_Ca_2.3 but is ineffective on K_Ca_3.1 channels [97].

For treating anosmia, COPD and its related pulmonary hypertension, K_Ca_2-positive modulators may be beneficial [42,46,62]. NS309 is a potent, non-selective activator of human K_Ca_3.1 and K_Ca_2 channels [98]. The K_Ca_2.2 and K_Ca_2.3 channels are potently and selectively activated by CyPPA [38], and their derivatives are chemically modified to create more efficient and selective positive modulators [99]. However, further investigations are needed to determine their effectiveness [33].

**Table 3:** Small-molecule positive and negative modulators of K_Ca_2 and K_Ca_3.1 channels.

	Nonselective K_Ca_2/K_Ca_3.1	K_Ca_2 Selective	K_Ca_3.1 Selective	Subtype K_Ca_2 Selective
**Positive modulators**	NS309 [98] SKA-31 [100] 1-EBIO [101] Riluzole [102]		SKA-111 [44] SKA-121 [103]	**K_Ca_2.2/K_Ca_2.3 selective**
				CyPPA [38] NS13001 [104] Compound 2q * [99]
				**K_Ca_2.1 selective**
				CM-TPMF [102]
**Negative** **modulators**	RA-2 [103]	NS5893 [104] AP14145 [97]	Senicapoc [11,95] TRAM-34 [96]	**K_Ca_2.1 selective**
				Bu-TPMF [102]

* 2q is a CyPPA-modified compound, other CyPPA modified compounds include: 2m–2n, 2p, 2r–2t, 2v, and 4. The potencies of these compounds on potentiating K_Ca_2.3 and K_Ca_2.2a channels have previously been determined [57,99].

## 6. Conclusions and Perspectives

In recent years, remarkable progress has been made in understanding the physiological and pathophysiological roles of K_Ca_ channels. The advances in understanding the K_Ca_3.1 structure and the resulting improvements in other K_Ca_2 subtypes modeling have yet to be used, not only for drug discovery but also for understanding the pathophysiological diseases, particularly airway diseases, and developing more subtype-selective biophysical and pharmacological tools. Over the past few years, researchers have studied K_Ca_3.1 channel expression and its physiological role in airway diseases. There are, however, few studies on K_Ca_2 channels in the respiratory system. Evidence now suggests that K_Ca_2 channels are present in the respiratory system and play an important role in airway disorders, such as asthma, chronic obstructive pulmonary disease, cystic fibrosis, and other muco-obstructive diseases. Nevertheless, further studies are necessary to unveil the exact cell distribution, subcellular localization, and protein interactions of K_Ca_2 channels in the airways. Additional research is required to further establish and validate K_Ca_2 and K_Ca_3.1 channels as ion channels in airway diseases, their clinical relevance, and the development of more potent and subtype selective K_Ca_2 channel modulators.

## Figures and Tables

**Table 1:** Apparent Ca^2+^ sensitivity, structural studies, amino acid sequences alignments and identities between K_Ca_2/3 channel subtypes.

K_Ca_2/3 α Subunit	Amino Acids	Apparent Ca^2+^ Sensitivity (μM)	K_Ca_2 Subtypes Structural Studies	Sequence Alignment among K_Ca_2 and K_Ca_3.1 Channels
K_Ca_2.1	543 [24]	~0.31 [25,26]	[27,28]	K_Ca_2.1 and K_Ca_3.1 share a 43.3% sequence identity [29]
K_Ca_2.2	579 [25]	~0.32 [25,30]	[15,26,29,31]	K_Ca_2.2 and K_Ca_3.1 share a 45% sequence identity [32]
K_Ca_2.3	731 [25]	~0.30 [33,34]	[34,35]	K_Ca_2.3 and K_Ca_3. share a 46.6% sequence identity [34]
K_Ca_3.1	427 [25]	~0.27 [33,34]	[36]	

**Table 2:** The *KCNN* gene family. Human chromosomal location, tissue distribution, functional effects, and their roles in the lungs.

K_Ca_2/3 α Subunit	Gene	Other Names	Human Chromosomal Location	Tissue Distribution	Physiological Roles	Role in the Lungs
K_Ca_2.1	*KCNN1*	SK1	19p13.11 [25]	Brain [25] Heart [43] Lung [41]	The K_Ca_2 channels underlie the medium AHP and regulate neuronal firing frequency [23,44].	ND *
K_Ca_2.2	*KCNN2*	SK2	5q22.3 [25]	Brain and heart Adrenal gland, lungs, prostate, bladder, and liver [25,45].		ND *
K_Ca_2.3	*KCNN3*	SK3	1q21.3 [25]	Brain and heart Vascular endothelium, lungs, and bladder [25,44]	K_Ca_2.3 and K_Ca_3.1 mediate the endothelium-derived hyperpolarization response [33,46]	(+) K_Ca_2.3 relaxes the pulmonary arteries and bronchi ** [32]
K_Ca_3.1	*KCNN4*	SK4 IK	19q13.31 [25]	Vascular endothelium T and B lymphocytes Microglia, placenta, colon, and red blood cells Lungs and bladder [25,44]	K_Ca_3.1 channels regulate calcium signaling, cellular activation, and cell volume [23,44]	(−) K_Ca_3.1 reduces Na^+^ absorption ***, (+) CBF, and MCC [5]. (+) K_Ca_3.1 relaxes the pulmonary arteries and bronchi [42]

* ND: not determined specifically in the respiratory system. ** (+): Activation. *** (−): Inhibition.

## Data Availability

Not applicable.

## Abbreviations

ASL	Airway surface liquid
Ca^2+^	Calcium
CaM	Calmodulin
Cl^−^	Chloride
COPD	Chronic obstructive pulmonary disease
CBF	Cilia beating frequency
K_Ca_	Ca^2+^-activated K^+^ channels
CF	Cystic fibrosis
CFTR	Cystic fibrosis transmembrane conductance regulator
K_Ca_3.1	Intermediate-conductance Ca^2+^-activated K^+^ channels
BK	Large-conductance Ca^+2^-activated K^+^ channels
MCC	Mucociliary clearance
ORNs	Olfactory receptor neurons
K^+^	Potassium
K_Ca_2	Small-conductance Ca^2+^-activated K^+^ channels
Na^+^	Sodium
TMs	Transmembrane helices
WT	Wild type
